# Inv(3) Acute Myeloid Leukemia in a Young Adult and Review of the Literature

**DOI:** 10.1155/2023/6628492

**Published:** 2023-11-11

**Authors:** Carlee Blakemore, Sudarshawn Damodharan, Diane Puccetti

**Affiliations:** ^1^Department of Pediatrics, University of Wisconsin School of Medicine & Public Health, Madison, WI, USA; ^2^Division of Pediatric Hematology, Oncology and Bone Marrow Transplant, University of Wisconsin School of Medicine & Public Health, Madison, WI, USA

## Abstract

Acute myeloid leukemia (AML) with the high-risk variant inv(3)/t(3;3) or t(3;3)(q21;26.2) is rarely seen in the pediatric and young adult population. It is associated with poor outcomes with ineffective therapeutic options. Here, we present a case of an 18-year-old female with treatment refractory inv(3) AML in whom remission was unable to be obtained. Better treatment options are needed given the increased resistance to traditional therapy this subtype portrays. Here, we review the literature on pediatric and young adult inv(3) AML along with newer therapeutic options.

## 1. Introduction

Acute myeloid leukemia (AML) is more common in the adult population, representing approximately 80% of all adult leukemias compared to 20% of childhood leukemias [1]. Survival rates for pediatric AML have drastically increased over the past few decades with improved supportive care along with more precise risk stratification and associated treatments [2]. However, this is not true for all as certain cytogenetic findings are considered high-risk (HR) given their inferior outcomes and resistance to conventional treatment. One HR AML variant is inv(3)(q21q26.2) or t(3;3)(q21;26.2), accounting for only 1-2% of all AML cases and sparsely seen in the pediatric population [3, 4]. It is classified by the World Health Organization (WHO) as an independent entity of AML and is also a prognostic marker for poor outcomes with resistance to standard chemotherapy [3, 5–7]. Here, we present a case of a young adult diagnosed with treatment refractory inv(3) AML. We also review the relevant literature to bring awareness of this subtype of AML that is rarely seen in the pediatric and young adult population.

## 2. Case Description

An 18-year-old female presented with prolonged fatigue and easy bruising. Her complete blood count was significant for anemia along with leukocytosis with the presence of peripheral blasts. Bone marrow aspirate demonstrated a hypocellular marrow, including 17% blasts. Flow cytometry was consistent with a myeloid phenotype (CD34+, CD117+). Cytogenetics were significant for inv(3)(q21q26.2);MECOM(EVI1). Next-generation sequencing (NGS) myeloid panel showed variants along with associated allelic frequencies (VAF) in NRAS (VAF 50%), WT1 (VAF 38%), and GATA2 (VAF 47%). No FLT3-TKD mutation was detected, with only weak FLT3-ITD positivity (ITD allelic ratio < 0.05). Our patient was diagnosed with HR inv(3) AML.

She received induction therapy per Children's Oncology Group (COG) protocol AAML1831, arm B, with intravenous (IV) liposomal cytarabine-daunorubicin (CPX-351) and gemtuzumab, on study. No central nervous system disease was present (CNS1). Disease assessment with repeat bone marrow aspirate revealed an increased presence of blasts up to 65% on morphology. She was taken off study and treated with a cycle of IV azacitidine and oral venetoclax (VEN/AZA). Repeat disease assessment showed continued presence of disease with 17% blasts via morphology and 7% via minimal residual disease (MRD). Another cycle of VEN/AZA was given. Repeat disease assessment once again showed persistent disease with 11% blasts via morphology and 3.6% via MRD. Continued salvage therapy was given with IV thiotepa, vinorelbine, topotecan, and clofarabine (TVTC). Disease assessment after this showed 20% blasts via morphology along with 6.3% via MRD.

Our patient overall tolerated these therapies well with minimal complications. Given the significant refractory nature of her disease, we discussed the poor prognosis. Our patient and her family elected to pursue continued therapy and are exploring clinical trial options with hope of ultimately being able to undergo a hematopoietic stem cell transplant (HSCT) for cure.

## 3. Discussion

Despite advancements in the understanding of AML biology, limited progress has been made in treatment outcomes for patients with HR AML, including inv(3) [1, 2, 4, 6]. This mutation results in a gene inversion which places the ecotropic viral integration site-1 (EVI1) gene, a highly conserved proto-oncogene, next to the ribophorin 1 (RPN1) gene and GATA2 enhancer, causing transcriptional activation of EVI1. This overexpression leads to increased myeloid cell proliferation as well as impaired cellular differentiation [3–5, 8–11]. Increased EVI1 expression in AML patients has been linked to poorer outcomes and treatment refractory disease, with inv(3) demonstrating the highest increases in EVI1 overexpression [12].

The median OS for patients with inv(3) AML is dismal with a large-scale study reporting a median OS of 10 months from diagnosis [5]. The poor prognosis is thought to be contributed by an increased resistance to chemotherapy causing an inability to achieve a complete remission (CR), as well as a high incidence of early relapse in those patients who do achieve this [4, 5, 11]. This variant is more commonly seen in adults and is exceedingly rare in pediatric patients [1, 3, 4]. Within the literature, only four cases of inv(3) AML have been reported in patients aged 0-18 years (Table 1) [11–14]. While the specific treatment and clinical course data are not available for the published cases, the treatment refractory nature of the inv(3) variant was highlighted.

Better treatments are needed for patients with inv(3) AML. Increased overall survival (OS) is seen in patients who are able to achieve a CR and proceed to a HSCT [15]. HSCT in HR AML has been associated with a longer median OS compared to standard chemotherapy alone. However, early relapse after HSCT has been well described in patients with inv(3) AML [4, 5, 10]. This data is confounded by the fact that patients are often transplanted with active disease present as part of their salvage regimen [4]. Given the increased chance for definitive cure with HSCT, efforts should be made to find treatments aimed at achieving a CR to facilitate a HSCT.

Alternative therapies that have been explored include targeting EVI1 transcriptional regulation given the high overexpression present in inv(3) AML. With the use of next-generation sequencing (NGS), additional mutations in RAS/RTK signaling pathways have also been detected alongside inv(3), indicating the possible application of FLT3 or PIK3 inhibition in these patients [4, 9]. One case showed achievement of CR in an adult patient with inv(3) AML following treatment with dasatinib, a tyrosine kinase inhibitor targeting AKT/STAT3, allowing for successful HSCT [14]. Hypomethylating agents (HMAs) such as azacitidine or decitabine have been used for many years in the treatment of AML, including the pediatric population [16, 17]. B cell lymphoma 2 (BCL-2) inhibitors, such as venetoclax, have been combined with HMAs in recent years as they have the potential to increase tumor sensitivity to HMAs, with further large-scale pediatric trials ongoing to assess sustained efficacy [17].

Developing immunotherapies for AML presents significant challenges including limited tumor-specific antigens and myeloid antigen heterogeneity [18, 19]. However, multiple targets have been explored including CD33, CD123, CLL-1, CD70, TIM-3, and folate receptor *α* and *β*. Clinical trials are ongoing to explore the efficacy of these modalities with the use of monoclonal and bispecific antibodies, antibody-drug conjugates, and chimeric antigen receptor (CAR) T cells (Table 2) [18, 19]. Additionally, trials are being conducted to combat pitfalls of immunotherapy in AML including minimizing toxicities, increasing efficacy, and overcoming resistance [18].

Pediatric and young adult inv(3) AML is a rare disease with a dismal prognosis. As with our patient, the hope is to achieve a CR and successfully undergo a HSCT for long-term cure, but this is not easily done. Continued research to find better treatment options is needed to better help this population.

## Figures and Tables

**Table 1:** Published cases of acute myeloid leukemia with the presence of inv(3) mutation in patients aged 0-18 years.

Case	Age at diagnosis (years)	Sex	Cytogenetics	Reference
1	17.8	M	45,XY,inv(3)(q21q26),-7	Haltrich et al. [11]
2	14	F	46,XX,inv(3)(q21q26)	Poppe et al. [12]
3	18	M	46,XY,inv(3)(q21q26),-7	Poppe et al. [12]
4	7	F	46,XX,inv(3)(q21q26),t(9;22)(q34;q11)/45,idem,-7	Behrens et al. [14]

Cases found on Mitelman Database of Chromosome Aberrations and Gene Fusions in Cancer as of 1 June 2023.

**Table 2:** Active immunotherapy clinical trials for pediatric and young adult AML.

Study title	NCT number	Therapeutic intervention	Country
First-in-human Study of SAR443579 Infusion in Male and Female Participants of at Least 12 Years of Age with Relapsed or Refractory Acute Myeloid Leukemia (R/R AML), B-cell Acute Lymphoblastic Leukemia (B-ALL) or High Risk- myelodysplasia (HR-MDS)	NCT05086315	Drug: SAR443579	USA, Australia, France, Netherlands
Phase I/II Study of Enhanced CD33 CAR T Cells in Subjects with Relapsed or Refractory Acute Myeloid Leukemia	NCT04835519	Biological: chimeric antigen receptor T cell	China
CLL-1/CD33 Targeted LCAR- AMDR Cells in Patients with Relapsed or Refractory Acute Myeloid Leukemia	NCT05654779	Biological: LCAR-AMDR Cells Product	China
Study of Anti-CD33 Chimeric Antigen Receptor-Expressing T Cells (CD33CART) in Children and Young Adults with Relapsed/Refractory Acute Myeloid Leukemia	NCT03971799	Biological: CD33CART	USA
Anti-FLT3 CAR T-cell Therapy in FLT3 Positive Relapsed/ Refractory Acute Myeloid Leukemia	NCT05023707	Biological: anti-FLT3 CAR-T	China
Anti-CLL1 CAR T-cell Therapy in CLL1 Positive Relapsed/ Refractory Acute Myeloid Leukemia (AML)	NCT04884984	Biological: anti-CLL1 CART	China
CD123-Targeted CAR-T Cell Therapy for Relapsed/Refractory Acute Myeloid Leukemia	NCT04265963	Biological: CD123 CAR-T cells	China
CD38-targeted Chimeric Antigen Receptor T Cell (CART) in Relapsed or Refractory Acute Myeloid Leukemia	NCT04351022	Biological: CART-38	China
Safety and Efficacy Study of CI-135 CAR-T Cells in Subjects with Relapsed or Refractory Acute Myeloid Leukemia	NCT05266950	Biological: CI-135 CAR-T cells	China
Safety and Efficacy of CD123- Targeted CAR-T Therapy for Relapsed/Refractory Acute Myeloid Leukemia	NCT04272125	Biological: CD123 CAR-T cells	China
PLAT-08: A Study Of SC- DARIC33 CAR T Cells in Pediatric and Young Adults with Relapsed or Refractory CD33+ AML	NCT05105152	Biological: SC-DARIC33	USA
CART-19 T Cell in CD19 Positive Relapsed or Refractory Acute Myeloid Leukemia (AML)	NCT03896854	Biological: CART-19	China
NKG2D CAR-NK & relapsed/refractory AML	NCT05734898	Biological: NKG2D CAR-NK	China
Safety and Preliminary Efficacy of JK500 Cell Injection in Relapsed/Refractory Pediatric Acute Myeloid Leukemia	NCT05519384	Drug: JK500 cell injection	China
Optimized Dual CD33/CLL1 CAR T Cells in Subjects with Refractory or Relapsed Acute Myeloid Leukemia	NCT05248685	Biological: Dual CD33/CLL1 CAR T	China
Multi-institutional Prospective Research of Expanded Multi- antigen Specifically Oriented Lymphocytes for the Treatment of Very High-Risk Hematopoietic Malignancies	NCT02203903	Biological: tumor-associated antigen lymphocytes	USA

Clinical trials found on http://www.clinicaltrials.gov as of 1 June 2023.

## Data Availability

Data sharing is not applicable to this article as no datasets were generated or analysed during the current study.

## Abbreviations

AML: Acute myeloid leukemia
CAR: Chimeric antigen receptor
COG: Children's Oncology Group
CR: Complete remission
HMA: Hypomethylating agent
HR: High-risk
HSCT: Hematopoietic stem cell transplant
MRD: Minimal residual disease
NGS: Next-generation sequencing
OS: Overall survival
VAF: Variant allelic frequency
WHO: World Health Organization.
